# Association of a Multimodal Educational Intervention for Primary Care Physicians With Prescriptions of Buprenorphine for Opioid Use Disorders

**DOI:** 10.1001/jamanetworkopen.2019.13818

**Published:** 2019-10-23

**Authors:** Brinton Clark, Mari Kai, Ryan Dix, Jonathan White, Yelena Rozenfeld, Sheldon Levy, Kathleen Engstrom

**Affiliations:** 1 Department of Medical Education, Providence Portland Medical Center, Portland, Oregon; 2 Providence Medical Group, Portland, Oregon; 3 Froedtert & Medical College of Wisconsin, Milwaukee; 4 Providence St Joseph Health, Portland, Oregon

## Abstract

**Importance:**

Opioid use disorder (OUD) is a public health crisis in the United States, but only 5% of US physicians have obtained a Drug Addiction Treatment Act (DATA) waiver to prescribe buprenorphine to treat OUD. Increasing the number of primary care physicians (PCPs) who have obtained the waiver and are able to treat patients with OUD is of utmost importance.

**Objective:**

To determine whether a multimodal educational intervention of PCPs is associated with an increase in the number of buprenorphine waivers obtained and patients initiated into treatment in a primary care setting.

**Design, Setting, and Participants:**

This quality improvement study was conducted in primary health care clinics within a large, integrated health care system. Patients included those who had received a diagnosis of OUD, and had Providence Health Plan Medicare or Medicaid insurance. Included PCPs were divided into 2 groups: those who obtained a DATA waiver after an education intervention (uptake PCPs) vs those who did not obtain a DATA waiver (nonuptake PCPs). The study took place between January 1, 2016, and December 31, 2017. Data analyses were conducted from December 2017 to August 2019.

**Exposures:**

Multimodal educational intervention including video, in-person visits to clinical practitioner meetings by physician champions, and a primary care toolkit with training resources and clinic protocols.

**Main Outcomes and Measures:**

The number of new uptake clinics where at least 1 PCP obtained a DATA waiver, the number of new PCPs with DATA waivers, the number of patients receiving a buprenorphine prescription, and the number of patients who received 12 or more weeks of treatment.

**Results:**

Twenty-seven of 41 invited clinics implemented the intervention, and 620 PCPs were included. The number of PCPs with DATA waivers increased from 5 PCPs (0.8%) to 44 PCPs (7.1%), and the number of clinics with at least 1 buprenorphine prescriber increased from 3 clinics (7.3%) to 17 clinics (41.5%). In total, 213 patients underwent buprenorphine treatment, and 140 patients received 12 or more weeks of treatment. A total of 646 patients had Providence Health Plan Medicare or Medicaid insurance and were eligible for the study (mean [SD] age, 61.7 [16.5] years; 410 [63.5%] women). There was a statistically significant difference in treatment with buprenorphine between patients with uptake PCPs vs patients with nonuptake PCPs (23 patients [16.4%] vs 18 patients [3.5%]; odds ratio, 4.61 [95% CI, 2.32-10.51]; *P* = .01) after the intervention.

**Conclusions and Relevance:**

In this quality improvement study, an educational intervention was associated with an increase in the number of PCPs and clinics that could provide buprenorphine treatment for OUD and with an increase in the patients who were able to access care with medications for OUD.

## Key Points

**Question:**

Is implementation of a multimodal educational intervention for primary care physicians (PCPs) associated with PCPs obtaining Drug Addiction Treatment Act waivers to prescribe buprenorphine to treat opioid use disorder (OUD) or with the number of patients with OUD who receive such treatment?

**Findings:**

In this quality improvement study, rates of treatment of OUD with buprenorphine were significantly higher after the intervention among patients with a PCP who had obtained a waiver after an educational intervention than among patients with a PCP who did not obtain a waiver.

**Meaning:**

A multimodal educational intervention with physicians in primary care clinics may increase the number of PCPs with buprenorphine waivers and the number of patients able to access buprenorphine treatment for OUD.

## Teaser

This quality improvement study examines the association of a multimodal educational intervention for primary care physicians (PCPs) with prescriptions of buprenorphine for treatment of patients with opioid use disorder.

## Introduction

Opioid use disorder (OUD) is considered a public health crisis in the United States, with steep increases in deaths attributable to natural and synthetic opioids in recent years.^1^ It is estimated that 2.1 million people in the United States have a substance use disorder related to prescription opioids, heroin, or both.^2^ The Drug Addiction and Treatment Act (DATA) of 2000 allows physicians to obtain a waiver to prescribe buprenorphine to treat OUD. Multiple studies have demonstrated that medications for OUD (MOUDs) decrease illicit opioid use and relapse, reduce risk behaviors for HIV infection, and reduce mortality in patients with OUD.^3,4,5,6^ Despite studies showing the clear efficacy of MOUD, only 21.5% of people with OUD in the United States receive treatment, which may be at least partially due to a limited number of physicians able and willing to prescribe MOUD.^7^

Given low rates of treatment of OUD, there is increasing interest in expanding MOUD more broadly into clinic-based settings where most patients receive their ongoing medical care.^8^ However, only 5% of US physicians have obtained a DATA waiver to prescribe buprenorphine, and only 27% of those have a waiver to treat more than 30 patients.^9^ Prior research has suggested that common barriers among physicians to prescribing buprenorphine include insufficient time, payment concerns, insufficient nursing or office staff support, concerns about diversion, lack of belief in opioid agonist therapy, insufficient physician or staff knowledge, and cumbersome regulations.^10,11,12,13^

This study was designed to engage, educate, and increase the number of primary care physicians (PCPs) trained and willing to treat patients using buprenorphine in their outpatient practices. We hypothesized that by reducing barriers and demystifying the process of MOUD, more PCPs would prescribe MOUDs in their practices and therefore increase the number of patients in treatment. This study was set at the Providence Medical Group (PMG), a large primary care network in Oregon within a larger multistate integrated health system.

## Methods

### Study Design

This quality improvement study examined the association of a multimodal educational intervention with increasing the number of PCPs who had obtained DATA waivers to prescribe buprenorphine and the number of patients treated with MOUDs. Outcomes of the intervention were reported at clinic, physician, and patient levels. The study was approved by the Providence Health and Services institutional review board. A waiver of informed consent was granted by the institutional review board because patient data were deidentified. This study was conducted in accordance with the Standards for Quality Improvement Reporting Excellence (SQUIRE) reporting guideline.

### Participant Sites

Providence Medical Group is a large primary care network within a larger multistate integrated health system. This study targeted PCPs in clinics that were part of the Portland, Oregon, service area of PMG, including regions from Hood River, Oregon; Newberg, Oregon; to Southwest Washington. The clinics reflected a variety of population, economic, and community resource differences and included internal medicine and family medicine PCPs. A total of 41 clinics were invited to participate, including 14 internal medicine clinics and 30 family medicine clinics. The clinics were patient-centered medical homes and incorporated psychologists and clinical pharmacists into their practices. The intervention team included 2 physician champions (B.C. and M.K.), a clinical pharmacist (J.W.), a psychologist (R.D.), and a project manager (K.E.).

### Patients

All included patients had received a diagnosis of OUD and had a PCP within PMG (eTable 1 in the Supplement). Owing to system limitations for extracting prescription data, we only included data from patients with Providence Health Plan insurance who were Medicare or Medicaid beneficiaries for our in-depth analysis. Patients were included if they had an OUD diagnosis between January 1, 2015, and December 31, 2017, accessed care in a PMG facility in the last 12 months, had at least 6 months of Providence Health Plan enrollment, had a PCP within PMG after January 1, 2016, and had complete insurance claims from the analysis period (eFigure in the Supplement).

### Intervention

The project’s educational intervention was introduced at regional clinic medical director and clinic manager meetings by the physician champions. Twenty-seven of 41 clinics agreed for the project team to meet with their physicians, nurse practitioners, and physician assistants during monthly clinic practice meetings. Some of the reasons cited for declining the intervention by the other 14 clinics were lack of time, not enough interest from the physicians, and that their clinic panel did not include many patients with OUD. An email was sent to an internal listserv of all PMG PCPs inviting them to participate in a baseline online survey. The survey was developed based on barriers previously identified in published literature^12,13^; included yes or no, Likert scale, and ranking multiple choice questions; and was assessed by an expert panel for clarity. The survey asked PCPs about knowledge and beliefs about buprenorphine, whether they would be likely to prescribe the medication, and about any barriers associated with prescribing buprenorphine for treatment of OUDs (eTable 2 in the Supplement). Initial survey results identified barriers to treatment of OUD with MOUD and were used to help formulate the educational interventions (eTable 3 in the Supplement). The educational intervention consisted of several elements. An approximately 10-minute educational video was developed that highlighted the experience of the physician champions in treating OUD in the primary care setting (eAppendix in the Supplement). The video explained the pharmacologic mechanisms of buprenorphine, discussed the advantages of treating with MOUD in the primary care setting, provided a brief overview of visit structure, shared success stories of patients treated by the physician champions, and addressed some of the barriers and concerns commonly voiced by PCPs about treating OUD with MOUD. A buprenorphine primary care toolkit was created with input from the physician champions, the pharmacist, and behaviorist and included training resources, suggested protocols, note templates, and suggested roles for behavioral health and clinical pharmacy practitioners. The toolkit was distributed to PCPs and clinical staff at the practice meetings and was made available on the PMG intranet site. The physician champions visited all of the clinics that participated in the intervention during practice meetings to discuss their experiences as PCPs in integrating MOUD to treat patients with OUD, address perceived barriers, and answer questions about their practice. In addition, a monthly newsletter describing recent research articles and press releases on topics related to OUD treatment was sent to interested PCPs. A 30-minute monthly conference call for case discussion and coaching was made available between the physician champions and participating PCPs. Ongoing 1-on-1 coaching was made available with the physician champions for new MOUD prescribers. Our grant reimbursed buprenorphine training, and an extra day designated for continuing medical education was offered to participants who underwent buprenorphine training.

### Outcomes

We examined a clinic-level outcome, 2 patient-level outcomes, and several physician-level outcomes. Physician- and clinic-level outcomes were measured using PCP surveys and direct follow-up with the PCPs, clinic managers, and credentialing office to track waivers. Patient-level outcomes were measured using electronic health records and insurance claims data to evaluate patients with OUD disorders, initiation of buprenorphine, and length of buprenorphine therapy. The initial survey asked PCPs to rank barriers to prescribing buprenorphine. At the clinic level, we measured the number of new clinics where at least 1 PCP received training and obtained a DATA waiver to prescribe buprenorphine and 1 or more patients received treatment for OUD with buprenorphine. The physician-level outcomes included the number of PCPs with DATA waivers. Cumulative waiver uptake per clinic and individual PCP DATA waiver information was obtained by the project manager (K.E.) via outreach to clinic managers, PCPs, and physician credentialing coordinators at our institution. The electronic health record data were used for the patient-level outcomes, defined as (1) the number of patients receiving a buprenorphine prescription during the study who had not received buprenorphine in the past year, and (2) the number of patients who received 12 or more weeks of treatment.

### Statistical Analysis

Descriptive statistics including proportions and means with SDs were used to summarize the results of the survey, patient demographics, and outcomes. We compared characteristics among patients with uptake PCPs vs those with nonuptake PCPs, including age, sex, and insurance using the *t* test for continuous variables and the χ^2^ test for proportions. Patients receiving care from the same PCP within the same practice were likely to reside in the same community and share other commonalities of the PCP prescribing pattern or practice characteristics. The effects of clustering were accounted for during statistical analysis. When comparing study groups for receipt of buprenorphine, we measured it at the individual patient level but adjusted for clustering effect to account for the association that naturally exists among patients from the same PCP. The Rao-Scott χ^2^ test, a cluster design-adjusted alternative of the Pearson χ^2^, was used to evaluate the difference between the study groups. The SURVEYFREQ procedure in SAS Enterprise Guide statistical software version 7.12 (SAS Institute) was used to complete this analysis. In addition, in this observational study without randomization or propensity matching to control for confounders, we used the SAS SURVEYLOGISTIC procedure to evaluate an intervention effect for the study outcome while adjusting for a clustering effect and controlling for baseline difference between the groups in mean age and insurance distribution. *P* values were 2-tailed, and statistical significance was set at less than .05.

## Results

Twenty-seven of 41 clinics (65.9%) agreed to implement the intervention, and 14 clinics (34.1%) did not agree to implement the intervention. A total of 620 PCPs were sent the survey and offered the opportunity for the educational intervention. The response rate to the initial survey was 16.5% (102 PCPs). Among 864 patients with OUD treated at the clinics, 646 patients (mean [SD] age, 61.7 [16.5] years; 410 [63.5%] women) met inclusion criteria. Among them, 362 patients had Medicare insurance (56.0%) and 284 patients had Medicaid insurance (44.0%). There was no statistically significant difference between the groups prior to the intervention with respect to prescribing buprenorphine (Table 1).

**Table 1.** Demographic Characteristics of Patients With Opioid Use Disorder Who Had Providence Health Plan Medicare or Medicaid

Characteristic	Patients, No. (%)			*P* Value
	Uptake PCP (n = 140)	Nonuptake PCP (n = 506)	Total (N = 646)	
PCP clusters, No.	29	109	138	NA
Age, mean (SD), y	57.3 (16.8)	62.9 (16.3)	61.7 (16.5)	<.001
Patients per PCP, mean (SD), No.	4.8 (4.7)	4.6 (4.9)	4.7 (4.9)	.85
Sex				
Men	52 (37.1)	184 (36.4)	236 (36.5)	.87
Women	88 (62.9)	322 (63.6)	410 (63.5)	
Insurance				
Medicare	57 (40.7)	305 (60.3)	362 (56.0)	<.001
Medicaid	83 (59.3)	201 (39.7)	284 (44.0)	
Received buprenorphine prescription before the intervention	2 (1.4)	5 (0.98)	7 (1.08)	.68
Received buprenorphine prescription after the intervention	23 (16.4)^a^	18 (3.5)^a^	41 (297%)	.01

Abbreviation: NA, not applicable; PCP, primary care physician.

^a^ Odds ratio, 4.61 (95% CI, 2.32-10.51); adjusted odds ratio, 4.17 (95% CI, 1.69-10.48); Rao-Scott χ^2^, 6.54.

The initial survey of PMG PCPs before the start of the educational intervention elucidated perceived barriers to prescribing buprenorphine. The top 3 barriers identified were a challenging patient population, limited outpatient counseling options for substance use or psychiatric comorbidities, and the time it takes to manage OUD treatment (eTable 3 in the Supplement). At the beginning of the study on January 1, 2016, 3 clinics (7.3%) had at least 1 PCP with a DATA waiver who was prescribing buprenorphine. At the end of the study on November 30, 2017, after our educational interventions, 17 clinics (41.5%) had at least 1 PCP with a DATA waiver who was prescribing buprenorphine. At study initiation, 5 PCPs (0.8%) had a DATA waiver. At the study’s end, 44 PCPs (7.1%) had a DATA waiver, representing nearly a 9-fold increase in PCPs with a DATA waiver (Table 2). During the study, 213 patients (33.0%) underwent buprenorphine treatment, and 140 patients (21.7%) achieved at least 12 weeks of treatment (Table 3).

**Table 2.** Physicians With DATA Waivers

Outcome	Patients, No. (%)	
	Preintervention	Postintervention
Clinics with ≥1 buprenorphine prescriber^a^	3 (7.3)	17 (41.5)
Physicians with a DATA waiver^b^	5 (0.8)	44 (7.1)

Abbreviation: DATA, Drug Addiction and Treatment Act of 2000.

^a^ Includes 41 clinics that participated in the intervention.

^b^ Includes 620 primary care physicians from 41 clinics.

**Table 3.** Patients With Any Insurance Type Undergoing Buprenorphine Treatment at All Clinics From 2016 Through 2017

Treatment	Patients, No.
Undergoing buprenorphine treatment	213
Treated with ≥12 wk of buprenorphine	140

Among 646 patients analyzed for patient-level outcomes, 140 patients (21.7%) had an uptake PCP and 506 patients (78.3%) had a nonuptake PCP (eFigure in the Supplement). There was no difference in buprenorphine prescribing between the groups during the preintervention period (Table 1). Prior to the intervention, only 7 patients (1.1%) were receiving MOUD treatment with buprenorphine. After the intervention, a total of 41 patients (6.4%) were prescribed buprenorphine during the study, including 23 patients with an uptake PCP (16.4%) and 18 patients with a nonuptake PCP (3.5%) (odds ratio, 4.61 [95% CI, 2.32-10.51]; *P* = .01). When logistic regression analysis was used to control for sex, age, and insurance status, results remained statistically significant (adjusted odds ratio, 4.17 [95% CI, 1.69-10.48]; *P* = .003) (Table 1).

## Discussion

Despite passage of DATA in 2000, a limited proportion of patients with OUD receive treatment with MOUD. While not the only contributor, uptake among PCPs has been low nationwide and is one area in which treatment access can be improved. This study was associated with a nearly 9-fold increase in the number of PCPs in the PMG who were trained and willing to treat OUD with buprenorphine. During the 2-year period of this study, nearly 215 patients underwent buprenorphine treatment. Additionally, our study demonstrated that patients with OUD whose PCP obtained a DATA waiver during the study had more than 4-fold greater odds of being treated with buprenorphine.

### Strengths and Limitations

This study had several strengths. First, potential barriers identified via survey were mitigated and addressed directly in the clinic outreach. The most important barrier was the perception of patients with OUD as challenging. The physician champions stressed the relative ease and rewards of treating this patient population during the clinic practice meetings and in the educational video. Second, buy-in and support from the regional leadership was obtained early on in the project. This included providing time for this education at the clinic practice meetings over other competing agendas and providing ongoing encouragement for PCPs to acquire a DATA waiver. Third, development of a treatment toolkit familiarized many PCPs with treatment processes and ways to use the medical home care team. One of the top 3 barriers that was identified via survey was the lack of behavioral health resources. The workflow that was created to use the behavioral health practitioners and clinical pharmacists who were integrated into the clinics may have lessened this perceived barrier. Finally, the personal, direct educational outreach by the physician champions, who were practicing PCPs rather than addiction specialists, may have helped to normalize the process.

This study also had limitations. First, not all clinics were willing to have the team visit to discuss the intervention. Additionally, some PCPs expressed interest in obtaining a DATA waiver but felt overwhelmed with other aspects of their practice and did not feel ready to take on a new challenge. Furthermore, new prescribers may have been slow to start providing treatment initially, limiting the potential for increased access to treatment. Hopefully, this concern will become less of an issue as PCPs become more comfortable through more experience. Another limitation of this study is that we did not have data on the demographics of PCPs who received their DATA waivers and started treating patients with MOUDs, so we were unable to determine which PCPs would be most likely to respond to this type of intervention. Our intervention was not randomized, and it is possible that clinics that accepted the educational intervention were different from clinics that declined the intervention in ways that were not captured by our study. It is similarly likely that PCPs who obtained their DATA waiver (uptake PCPs) were also different from PCPs who did not obtain their DATA waiver (nonuptake PCPs) in ways we were not able to analyze. Additionally, a more in-depth analysis of the likelihood of receiving treatment with buprenorphine was limited to patients with either Providence Health Plan Medicare or Medicaid insurance. This may have limited the generalizability of our results to patients with other types of insurance.

## Conclusions

This quality improvement study of a multilevel educational intervention during an 18-month period to improve access to opioid treatment in the primary care setting included a targeted PCP survey to identify barriers, an educational outreach via regional leadership, direct face-to-face meetings with PCPs, and provision of educational toolkits. The intervention was associated with an increase in the number of clinics and the number of prescribers in a large primary care network that could provide MOUD, with 213 patients starting MOUD during the intervention period. Other integrated health systems may consider multipronged educational outreach from medical home PCPs to increase the numbers of PCPs able and willing to treat OUDs with MOUDs.

## Notes

Supplement. **eTable 1.** List of Diagnoses Used to Define Opioid Use Disorder **eFigure.** Uptake vs Nonuptake Primary Care Providers (PCPs) and Receipt of Medication for Opioid Use Disorder (MOUD) **eTable 2.** Initial Physician Survey **eTable 3.** Physician Survey Results **eAppendix.** Intervention Educational Video
