# Supplementary material for: Association of a Multimodal Educational Intervention for Primary Care Physicians With Prescriptions of Buprenorphine for Opioid Use Disorders
Source: JAMA Netw Open. 2019 Oct 23;2(10):e1913818. doi: 10.1001/jamanetworkopen.2019.13818 (PMC6820030; doi:10.1001/jamanetworkopen.2019.13818)
Supplement: Supplement. — eTable 1. List of Diagnoses Used to Define Opioid Use Disorder eFigure. Uptake vs Nonuptake Primary Care Providers (PCPs) and Receipt of Medication for Opioid Use Disorder (MOUD) eTable 2. Initial Physician Survey eTable 3. Physician Survey Results eAppendix. Intervention Educational Video [file jamanetwopen-2-e1913818-s001.pdf]

Supplementary Online Content

Clark B, Kai M, Dix R, et al. Association of a multimodal educational intervention for primary care physicians with prescriptions of buprenorphine for opioid use disorders. *JAMA Netw Open*. 2019;2(10):e1913818. doi:10.1001/jamanetworkopen.2019.13818

eTable 1. List of Diagnoses Used to Define Opioid Use Disorder

eFigure. Uptake vs Nonuptake Primary Care Providers (PCPs) and Receipt of Medication for Opioid Use Disorder (MOUD)

eTable 2. Initial Physician Survey

eTable 3. Physician Survey Results

eAppendix. Intervention Educational Video

This supplementary material has been provided by the authors to give readers additional information about their work.

© 2019 Clark B et al. *JAMA Network Open.*

eTable 1. List of Diagnoses Used to Define Opioid Use Disorder*:

ICD Code	Description
F1110	Opioid abuse, uncomplicated
F11129	Opioid abuse with intoxication, unspecified
F11188	Opioid abuse with other opioid-induced disorder
F1119	Opioid abuse with unspecified opioid-induced disorder
F1120	Opioid dependence, uncomplicated
F1121	Opioid dependence, in remission
F11220	Opioid dependence with intoxication, uncomplicated
F1123	Opioid dependence with withdrawal
F1124	Opioid dependence with opioid-induced mood disorder
F11282	Opioid dependence with opioid-induced sleep disorder
F11288	Opioid dependence with other opioid-induced disorder
F1129	Opioid dependence with unspecified opioid-induced disorder
F1190	Opioid use, unspecified, uncomplicated
F11921	Opioid use, unspecified with intoxication delirium
F1193	Opioid use, unspecified with withdrawal
F11982	Opioid use, unspecified with opioid-induced sleep disorder
F11988	Opioid use, unspecified with other opioid-induced disorder
F1199	Opioid use, unspecified with unspecified opioid-induced disorder

*We included multiple related ICD10 codes to broadly capture patients with OUD.

© 2019 Clark B et al. *JAMA Network Open.*

eFigure. Uptake vs Nonuptake Primary Care Providers (PCPs) and Receipt of Medication for Opioid Use Disorder (MOUD)

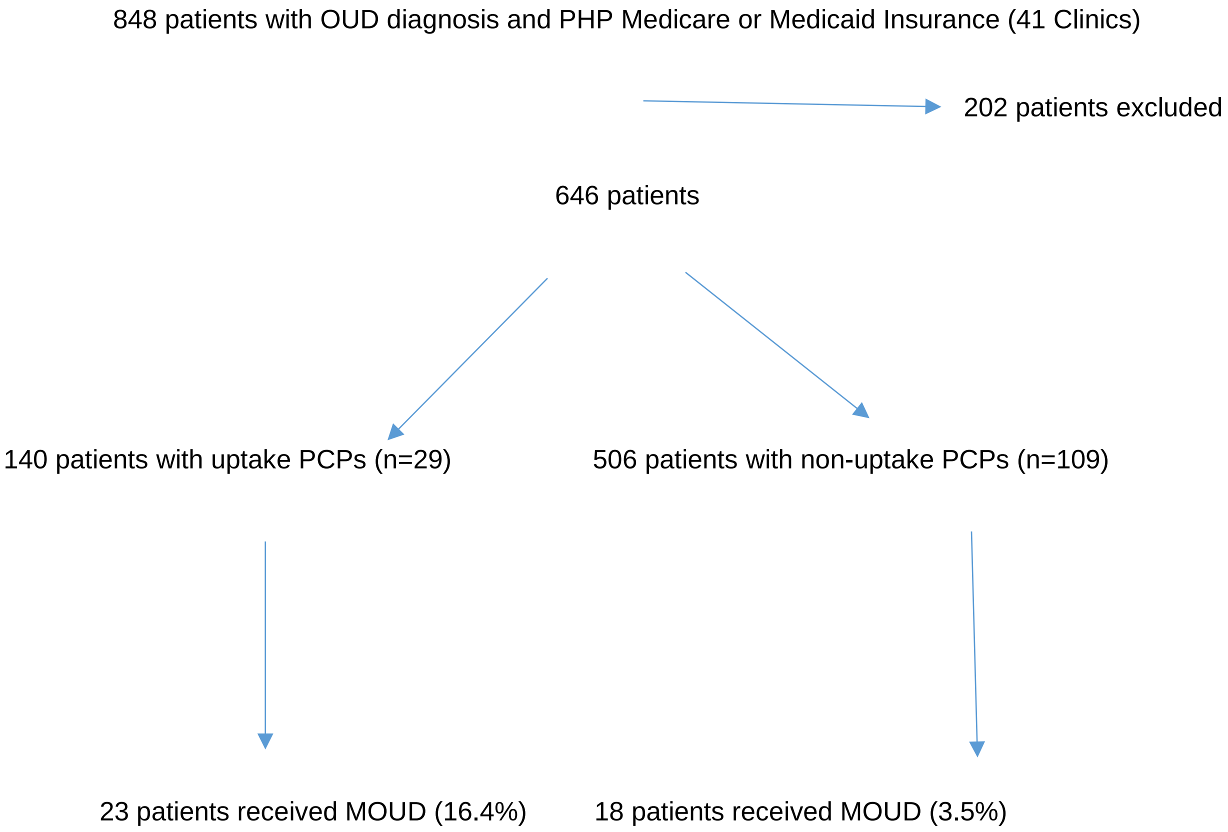

© 2019 Clark B et al. *JAMA Network Open.*

eTable 2. Initial Physician Survey

1. Do you know what Buprenorphine (Suboxone) is? Y or N
2. Do you personally think Buprenorphine should be provided for patients in primary care? Y or N?
3. Do you currently have the Buprenorphine DEA waiver to treat opiate addicted patients? Y or N?
4. Are you currently treating patients with Buprenorphine? Y or N?

   4a. If yes, approximately how many? _____patients

   4b. If no, how likely are you to incorporate Buprenorphine prescribing into your personal practice?

Very Unlikely	Unlikely	Somewhat Unlikely	Undecided	Somewhat Likely	Likely	Very Likely
1	2	3	4	5	6	7

5. Please rank the top 3 barriers you have to prescribing Buprenorphine

Concerns about effectiveness of Buprenorphine	
Difficult to titrate	
Adverse events	
Medical complications	
Concerns about diversion and abuse	
Challenging patient population	
Limited behavioral medicine support	
Limited pharmacy support	
Limited outpatient counseling options for substance use and/or psychiatric comorbidities	
Record-keeping requirement	
DEA involvement	
Reimbursement concerns	
Time it takes to manage	
Federal limits on number of patients	
Low demand	
Insufficient personal knowledge about prescribing Buprenorphine	
Other (please list)	

© 2019 Clark B et al. *JAMA Network Open.*

eTable 3. Physician Survey Results

	N (out of 102)	%
Challenging patient population	54	52%
Limited outpatient counseling options for substance use and/or psychiatric co-morbidities	52	51%
Time it takes to manage	31	30%
Insufficient personal knowledge about prescribing buprenorphine	29	28%
Concerns about diversions and abuse	23	23%
Limited behavioral medicine support	17	17%
Low Demand	11	11%
Adverse events	6	6%
Medical Complications	5	5%
DEA Involvement	5	5%
Concerns about effectiveness of buprenorphine	4	4%
Limited pharmacy support	4	4%
Record-keeping requirement	3	3%
Difficult to titrate	2	2%
Federal limits on number of patients	1	1%
Reimbursement concerns	0	0%

© 2019 Clark B et al. *JAMA Network Open.*

eAppendix. Intervention Educational Video

Link: https://youtu.be/OBHAgLIMIc4

Practitioner educational video highlights the experience of the physician champions in treating OUD in the primary care setting. Topics covered include the pharmacologic mechanisms of buprenorphine, the advantages of treating patients with OUD in the primary care setting, visit structure and success stories of patients with OUD treated by the physician champions.

© 2019 Clark B et al. *JAMA Network Open.*
